# Anti-Inflammatory and Vasoprotective Activity of a Retroviral-Derived Peptide, Homologous to Human Endogenous Retroviruses: Endothelial Cell Effects

**DOI:** 10.1371/journal.pone.0052693

**Published:** 2012-12-20

**Authors:** George J. Cianciolo, Salvatore V. Pizzo

**Affiliations:** Department of Pathology, Duke University Medical Center, Durham, North Carolina, United States of America; Brigham and Women's Hospital, United States of America

## Abstract

Malignant and inflammatory tissues sometimes express endogenous retroviruses or their proteins. A highly-conserved sequence from retroviral transmembrane (TM) proteins, termed the “immunosuppressive domain (ID)”, is associated with inhibition of immune and inflammatory functions. An octadecapeptide (MN10021) from the ID of retroviral TM protein p15E inhibits *in vitro* release of pro-inflammatory cytokines and increases synthesis of anti-inflammatory IL-10. We sought to determine if MN10021 has significant *in vivo* effects. MN10021, prepared by solid-phase synthesis, was dimerized through a naturally-occurring, carboxy-terminal cysteine. *In vivo* anti-inflammatory activity was determined using a murine model of sodium periodate (NaIO_4_)-induced peritonitis. *In vivo* vasoprotective effects were determined using: (1) a carrageenan-induced model of disseminated intravascular coagulation (DIC) in mice; (2) a reverse passive Arthus model in guinea pigs; and (3) vasoregulatory effects in spontaneously hypertensive rats (SHR). *In vitro* studies included: (1) binding/uptake of MN10021 using human monocytes, cultured fibroblasts, and vascular endothelial cells (VEC); (2) gene expression by RT-PCR of MN10021-treated VEC; and (3) apoptosis of MN10021-treated VEC exposed to staurosporine or TNF-α. One-tenth nmol MN10021 inhibits 50 percent of the inflammatory response in the mouse peritonitis model. Furthermore, 73 nmol MN10021 completely protects mice in a lethal model of carrageenan-induced DIC and inhibits vascular leak in both the mouse DIC model and a guinea pig reverse passive Arthus reaction. MN10021 binds to and is taken up in a specific manner by both human monocytes and VEC but not by cultured human fibroblasts. Surprisingly, orally-administered MN10021 lowers blood pressure in SHR rats by 10–15% within 1 h suggesting a direct or indirect effect on the vascular endothelium. MN10021 and derived octapeptides induce iNOS (inducible nitric oxide synthase) mRNA in VEC and nitrate in VEC cell culture supernatants and protect VEC from induced apoptosis or necrosis. However, pretreatment of VEC with nitro-L-arginine methyl ester (L-NAME), while inhibiting the release of nitrate, does not block the anti-apoptotic effect of MN10021 and derived octapeptides suggesting that their potent vasoprotective and anti-inflammatory activity is not nitric oxide dependent.

## Background

We previously reported that fluids from patients representing 15 different types of neoplasms contained proteins which were capable of inhibiting the *in vitro* responses of human peripheral blood-derived monocytes to chemotactic agents and that these anti-inflammatory proteins were antigenically related to the retroviral transmembrane protein p15E [1]. We also demonstrated that both serially-passaged and spontaneous murine tumors, as well as the plasma and urine of mice bearing spontaneous tumors, contained p15E-related anti-inflammatory proteins [2] and that p15E purified from the transmembrane proteins of various retroviruses inhibited inflammation in mice [3]. We subsequently identified a 26-amino acid region of p15E which is highly conserved in not only murine retroviruses, but also feline, bovine, avian, and primate retroviruses as well as the human retroviruses HIV and HTLV (human T-lymphocyte leukemia) [4]. A synthetic peptide (CKS-17) corresponding to the first 17 amino acids of this conserved region of p15E, when conjugated to a carrier protein (BSA [bovine serum albumin]or HSA [human serum albumin]), inhibits a variety of immunological functions such as proliferation in response to mitogens or alloantigens [5]; production of cytokines such as TNF-α and IFN-γ [6], [7], [8]; production of superoxide anion [9]; NK cell activity [10]; polyclonal B cell activation [11]; generation of CTL activity [12]; and IL-1 mediated signal transduction [13]. CKS-17 also inhibits cell-mediated immunity *in vivo* [14]. This highly conserved 26-amino acid region of the retroviral transmembrane proteins is termed the “immunosuppressive region or domain” based on the inhibitory profile of CKS-17.

MN10021 is a homodimeric octadecapeptide, corresponding to the 17-amino acid sequence of CKS-17 with an additional (naturally-occurring) carboxy-terminal cysteine through which homodimers are readily formed. MN10021 has biological activities without any requirement for conjugation to a carrier protein such as was required for the monomeric CKS-17 peptide [15], [16], [17], [18]. In the present study we demonstrated that MN10021 retains the *in vitro* immunosuppressive/anti-inflammatory profile of CKS-17 and that MN10021 also has significant *in vivo* immunosuppressive/anti-inflammatory activity. The results of these studies and an unexpected result from a pharmacological profiling of MN10021 led us to examine vascular endothelial and smooth muscle cells as potential targets for MN10021 and related peptides. Our data suggest that while the ability of these peptides to induce vasorelaxant molecules such as nitric oxide (NO) may have therapeutic benefits, the potent anti-inflammatory activity of these peptides is probably unrelated to their ability to produce vasoprotective molecules such as NO.

## Methods

### Ethics Statement

All studies on human leukocytes were performed on cells obtained from healthy volunteers by Dr. Carlo DeCastro at Duke University under a Duke University Institutional Review Board (IRB)-approved protocol. All subjects read, signed and dated an Informed Consent Form, previously approved by the Duke University Institutional Review Board, and this signed consent was then witnessed and kept in a locked file by Dr. DeCastro according to the procedures approved by the Duke IRB. All mouse studies were carried out in accordance with protocols approved by the Institutional Animal Care and Use Committee (IACUC) at MacroNex Inc. at their facilities in Morrisville, NC. All guinea pig studies were carried out in accordance with protocols approved by the Institutional Animal Care and Use Committee (IACUC) at Pharmakon USA at their facilities in Waverly, PA. All rat studies were carried out in accordance with protocols approved by the Institutional Animal Care and Use Committee (IACUC) at PanLabs Inc., Bothell, WA at their facilities.

### Cells and Reagents

Peripheral blood mononuclear cells (PBMC) and polymorphonuclear leukocytes (neutrophils) were prepared in the following manner. Citrated (10% v/v; acid-citrate dextrose [ACD]; Sigma, St. Louis, MO) human blood was obtained from healthy volunteers under informed consent and mixed with an equal volume of sterile, isotonic saline. Approximately 30 ml of the diluted blood in a 50-ml conical polypropylene centrifuge tube was underlaid with 20 ml of Histopaque®-1077 (Sigma, St. Louis, MO) and the tubes centrifuged for 40 min at 22°C and 400×g. The mononuclear cells at the plasma-Histopaque® interphase were removed, washed 2X with sterile phosphate-buffered saline, pH 7.4 (PBS), washed once with Hanks Balanced Salt Solution (HBSS) or media (RPMI-1640), depending on their intended use, and then resuspended in either buffer or complete media. Neutrophils were isolated from the pellet of the density gradient separation by rapid (<20 s) hypotonic disruption of contaminating erythrocytes with 0.2% NaCl which was then made isotonic by addition of an equal volume of 1.6% NaCl. The lysis procedure was repeated once and the cells washed twice and resuspended in HBSS containing 10 mM HEPES, 0.1% BSA (low endotoxin; Sigma, St. Louis, MO), pH 7.2.

Pooled human umbilical vein endothelial cells (HUVEC; Clonetics®; Cambrex, East Rutherford, NJ) were obtained from and cultured according to the provided instructions using media and supplements obtained from Cambrex. Cells were generally used between passages 3 and 6. Human umbilical artery smooth muscle cells (HUASMC; Clonetics®; Cambrex, East Rutherford, NJ) were obtained from and cultured according to the provided instructions using media and supplements obtained from Cambrex. WI-38 human fibroblast cells (CCL-75™) were obtained from the ATCC (Manassas, VA) and grown according to ATCC supplied instructions.

Dexamethasone, acetaminophen, diclofenac, carrageenan, sodium periodate (NaIO_4_), cytochalasin B, nitro-L-arginine methyl ester (L-NAME), and SQ 22536 were all obtained from Sigma-Aldrich, St. Louis, MO.

### Media

Media for human PBMC culture was as follows: RPMI-1640 was supplemented with 100 u/ml penicillin, 100 µg/ml streptomycin, 2 mM L-glutamine, 1 mM Na pyruvate, 1% MEM non-essential amino acids, 25 mM HEPES (all from Sigma, St. Louis, MO) and 1% Nutridoma-HS (Boehinger Mannheim; Indianapolis, IN). Complete Medium is media supplemented with 5% heat-inactivated (56°C, 30 min) pooled human AB serum (Pel-Freez; Brown Deer, WI).

### Synthetic Peptides

MN10021, a dimerized octadecapeptide, and MN20050, its reversed sequence control, were first prepared by solid-phase synthesis and reverse-phase HPLC (RP-HPLC) purification of the corresponding monomer peptides: NH_2_-LQNRRGLDLLFLKEGGL-COOH and NH_2_-LGGEKLFLLDGRNQLC-COOH. The monomer peptides were allowed to spontaneously dimerize overnight by slow stirring at pH 9 and the resultant dimer peptides separated from monomer peptides by RP-HPLC. All peptides were >97% pure by reverse phase-HPLC and the correct molecular mass verified by mass spectroscopy. An additional peptide, MN20054, was synthesized with the phenylalanine (Phe) at position 11 replaced by a tyrosine (Tyr) to allow radiolabeling with [^125^I]. This peptide was purified, dimerized, and re-purified as described for MN10021 and MN20050 above. Monomer peptide analogs of MN10021 were prepared by solid phase synthesis and revere phase-HPLC purification by SynPep, CA. The following peptides were used: DUK0001, NH_2_-GLDLLFLK-COOH; DUK0004, acetyl-GLDLLFLK-acetyl; DUK0005, acetyl-GLDLLFLK-NH_2_; DUK0006, NH_2_-GLDLLFLK-NH_2_; DUK0007, acetyl-GLDLLYLK-NH_2_.

### Animals

Mice (CD-1) and guinea pigs (Hartley) were obtained from Charles River, Raleigh, NC, and Newfield, NJ and housed under NIH-approved standards. All studies were conducted under Institutional Animal care and Use Committee-approved protocols (MacroNex Inc., mice; Pharmakon USA, guinea pigs; and MDS-Panlabs, rats) with appropriate veterinary supervision.

### Assays of Peptide Binding/Uptake

An analog (MN20054) of MN10021 was synthesized with a Tyr11 in place of the Phe11 and subsequently radiolabeled with [^125^I] (Perkin Elmer; Boston, MA) using the chloramine-T method. Human blood monocytes (purified by adherence to plastic; 10^6^/0.2 ml in PBS with 0.5% BSA, pH 7.5 in 12×75 mm polypropylene tubes) were incubated in triplicate at 37°C for 60 min with 40 pmol [^125^I]-MN20054 (∼20 Ci/mmol) in the presence or absence of a 100-fold molar excess of unlabeled peptide. Tubes were shaken gently during the incubation. The contents of each tube were layered onto a layer of 0.8 ml of 10% sucrose in polypropylene Eppendorf tubes and the tubes centrifuged for 5 min at 8,000×g to separate cell-associated from free ligand. The tubes were then snap-frozen in a dry-ice/acetone bath and the bottom of the tube containing the cell pellet cut off and then counted in a gamma-scintillation spectrophotometer. In studies to determine how much of the uptake was due to endocytosis, binding/uptake was performed as described above with the exception that half of the monocytes were pretreated for 30 min at 37°C with cytochalasin B (10 µg/ml; Sigma; St. Louis, MO).

For binding/uptake studies on endothelial cell, HUVEC were grown to confluence in 12-well tissue culture plates. Forty pmol of [^125^I]-MN20054 (∼20 Ci/mmol) in PBS with 0.5% BSA, pH 7.5, was added in a volume of 1.0 ml in the presence or absence of a 100-fold excess of the indicated competitor. The plates were allowed to incubate for 1 h at 37°C on a rocking platform and the wells were aspirated and washed 3X with 2.0 ml of ice-cold PBS/BSA. One ml of 1.0 N NaOH was added to each well for 30 min at RT to solubilize the cells and then 0.9 ml was removed from each well and counted in a gamma scintillation spectrophotometer.

For binding/uptake studies on fibroblasts, WI-38 fibroblasts (CCL-75™; ATCC) were grown to confluence in 12-well tissue culture plates. To each of triplicate wells was added 20 pmol of [^125^I]-MN20054 in the presence of either buffer alone, a 100-fold molar excess of MN20054, or a 100-fold molar excess of MN10021. The plates were incubated for 60 min at 37°C with gentle rocking, the wells aspirated and quickly washed 3X with 2.0 ml each of ice-cold PBS/BSA and 1.0 ml of 1.0 N NaOH added to each well to solubilize the cells. Nine-tenths ml of solubilized cells was then removed from each well and cell-associated radioactivity determined by gamma scintillation spectrophotometry.

### Mouse Inflammatory Peritonitis Model

Effects of test compounds on the inflammatory accumulation of monocytes/macrophages or neutrophils *in vivo* were determined using a mouse peritonitis model. Briefly, groups of five 6–8 week old CD-1 outbred mice (Charles River; Raleigh, NC) were injected intraperitoneally (i.p.) with 1.0 ml of a sterile inflammatory stimulus (NaIO_4_; 5 mM in sterile isotonic saline; Sigma; St. Louis, MO). This procedure results in a dramatic accumulation of neutrophils in the peritoneal cavity which peaks at 6–8 h post injection, followed by an accumulation of monocytes/macrophages which peaks at 48–72 h post injection. Mice were euthanized by CO_2_ asphyxiation at either 6 h (for neutrophils) or 48 h (for monocyte/macrophages), the peritoneal cavities lavaged with two 10-ml washes of heparinized (10 u/ml; Sigma; St. Louis, MO) HBSS containing 0.1% (w/v) BSA, the washes pooled, and total and differential cells counts determined for each pooled cell suspension. Each test material was evaluated in a group of five mice, each of which was injected subcutaneously in the thigh with 0.2 ml of test material in HBSS 24 h prior to the i.p. injection of the inflammatory stimulus.

### Mouse Carrageenan-induced DIC Model

Groups of 10 male CD-1 mice were injected i.p. with 0.25 ml of either vehicle (PBS) or peptide in vehicle at varying times prior to the i.p. injection of 1.0 ml of carrageenan (0.45% [w/v] in PBS; Sigma). In experiments where serum cytokine levels were determined, groups of 5 mice were euthanized at 1-, 2-, 4-, 8-, and 24-h post injection of carrageenan, blood collected by cardiac puncture into heparinized syringes, and the resultant plasma stored at −80°C until assayed by ELISA. In the single carrageenan-induced DIC experiment where blood cell counts were determined, groups of 20 CD-1 mice were injected 18 h prior to an i.p. injection of carrageenan with either 1.0 ml i.p. saline or 1 mg (245 nmol) i.p. injected MN10021. At 1-, 2-, 4-, and 24-h post injection of carrageenan, five mice from each group were euthanized and heparinized blood samples collected from the heart using heparinized syringes for subsequent determinations of WBC (white blood cell) counts, RBC (red blood cell) counts, or PLT (platelet) counts.

### Vascular Leak Assays

Vascular permeability in the mouse carrageenan-model of DIC was determined by measuring extravasation of [^125^I]-BSA from the blood into the lungs. Groups of 5 CD-1 mice were pretreated with the indicated doses of MN10021 or vehicle (saline) injected i.p. 18 h prior to i.p. injection of carrageenan or saline. Twelve hours later the mice were each injected i.v. with 0.25 ml of saline containing 10^6^ cpm of [^125^I]-BSA followed by an i.p. injection of 1.0 ml of either saline or 0.45% carrageenan. Twelve hours after i.p. injection of carrageenan or saline the mice were euthanized, the lungs collected, radioactivity determined by gamma scintillation spectrophotometry, and the percent increase in lung-associated radioactivity determined by comparing the radioactivity in the lungs of the carrageenan-injected animals with that in the lungs of the mice injected i.p. with saline. Twelve hours post carrageenan injection was chosen for measurement of vascular leak because experience had indicated that no deaths in the untreated groups would occur earlier than this time.

Vascular permeability was assessed in the reverse passive Arthus reaction as follows: Groups of 5 male Hartley guinea pigs were injected s.c. with 62.5 to 500 µg (15–61 nmol) of MN10021 or vehicle (saline). Eighteen hours later both flanks of the animals were shaved and 6.25 or 12.5 µl of rabbit anti-BSA antiserum was injected intradermally (i.d.) at two sites, one on each flank. The animals were then injected i.v. with 1 ml of PBS containing BSA (1 mg/ml) and Evans Blue dye (2 mg/ml; Sigma; St. Louis, MO). Six hours later the animals were euthanized, the skin removed, and the level of induration determined as the diameter (mm) of the extravasation for each site measured on the ventral surface of the skin.

### SHR Rat Hypertension Model

As part of a general pharmacological screen, MN10021 was evaluated in a rat model of hypertension by MDS-PanLabs (Bothell, WA). Briefly, groups of 3 SHR (spontaneous hypertensive) rats were dose orally with either MN10021 (30 or 100 mg/kg; 7.3–24 µmol/kg) or, as a positive control, L-methyldopa (100 mg/kg; 474 µmol/kg). Blood pressure measurements were made by tail cuff at 1, 2, and 4 h post administration of test compounds. A decrease of ≥10% of the baseline systolic blood pressure is considered significant in this assay.

### RT-PCR

Cells were cultured with various concentrations of test materials, either with or without stimulus, for varying times. The cells were collected, washed and total RNA isolated using the RNeasy Mini Kit (Qiagen; Valencia, CA). Following isolation the RNA was treated with 7 units of DNase I according to the RNase-Free DNase Set (Qiagen; Valencia, CA) protocol. Using these procedures we usually obtained ∼50 µg of total RNA/10^7^ cells. cDNA was prepared from the isolated mRNAs and RT-PCR performed on the target cytokines/chemokines/growth factors using appropriate primer pairs and a control primer pair for β-actin (all from R & D Systems; Minneapolis, MN). RT-PCR was generally performed for 30 cycles for β-actin and 35–45 cycles for iNOS and VEGF.

### Apoptosis Assays in HUVEC

Apoptosis was measured in HUVEC using the Cell Death Detection ELISA^PLUS^ kit (Roche Diagnostics; Indianapolis, IN) according to the manufacturer's instructions. This assay is based on a quantitative sandwich-enzyme-immunoassay using mouse monoclonal antibodies directed against both DNA and histones, respectively. It allows the specific determination of mono- and oligonucleosomes in the cytoplasmic fraction of cell lysates (apoptosis) and in the supernatant of dying cells (necrosis). The apoptotic index (*AI*) was calculated as

where treatment refers to use of a known apoptosis- or necrosis-inducing agent (staurosporine, TNF-α).

### Assay for NO Release

HUVEC were grown to 80–90% confluence in 12-well tissue culture plates using defined media provided by Cambrex and supplemented with 10% FBS. Triplicate wells were incubated with either media alone or media containing 50 µM of the indicated peptide. Supernatant samples were removed from each well at 6 h and 24 h post peptide addition and assayed for NO release as measured by the presence of nitrate (converted to nitrite) using a kit supplied by Oxford Biomedical Research (Rochester Hill, MI) according to the manufacturer's instructions.

### Statistical Analysis

Statistical analysis was performed using a Student's *t* test. Values of p<0.05 were considered significant.

## Results

### *In Vitro* Anti-inflammatory Profile of MN10021

MN10021 inhibits the production or blocks the activity of a number of potential inflammatory mediators such as TNF-α, IL-1, IFN-γ and IL-2 (6–8, 13, 15). However, there has never been a systematic examination of the *in vitro* effects of MN10021 on human blood leukocytes in which many of these potentially-important, pro-inflammatory biological activities were examined on the same set of cells. We therefore isolated various human blood leukocytes from a large volume of blood and tested MN10021 and its control peptide, MN20050, at concentrations ranging from 10 nM to 100 µM. The results from a representative set of experiments are summarized in Table 1, in which the IC_50_ for MN10021 on various proinflammatory functions is listed. Since all the studies for each set were performed on blood cells from the same volunteer, we believe they provide a truly representative profile of the potential anti-inflammatory capabilities of MN10021. Similar results in these same assays were obtained on at least three additional occasions. MN20050, the reversed-sequence control peptide, had no reproducible inhibitory effect in any of these assays.

**Table 1:** Pharmacological Profile of MN10021.

Mediator/Function*	MN10021	MN20050
	IC_50_ (µM)	
TNF-α	6.2	NE
IL-1β	17	NE
IL-6	NE	NE
GM-CSF	8	NE
LTB_4_	6.2	NE
PAF	12.5	NE
TF	12.5	NE
Chemotaxis	0.25	NE
Adhesion	NE	NE
Platelet Aggregation	5	NE

NE  =  No Effect.

* Methods used described in Methods S1.

### Effect of MN10021 in a Mouse Model of Inflammatory Peritonitis

The *in vitro* profile of MN10021 suggested that it might have useful anti-inflammatory activity *in vivo*. The retroviral protein from which MN10021 is derived was first characterized as having anti-inflammatory activity in a mouse model of peritonitis induced by i.p. injection of an inflammatory stimulus [3]. As shown in Table 2, MN10021 has potent anti-inflammatory activity in this model in which the inflammatory accumulation of monocytes/macrophages is quantitated 48 h after i.p. injection of NaIO_4_, an oxidant known to induce an inflammatory peritoneal exudate [19]. MN10021 achieved a maximal 70% inhibition of inflammation at a dose of 4 µg (1 nmol) with an IC_50_ of 0.4 µg (0.1 nmol). MN20050, the control peptide, had no anti-inflammatory activity even at doses as high as 40 µg (10 nmol). The potency of the *in vivo* anti-inflammatory effects of MN10021 is illustrated by comparison of its activity with those of the steroid dexamethasone or the non-steroidal anti-inflammatory drugs acetaminophen or diclofenac. These three these agents achieved somewhat similar or slightly higher maximal levels of inhibition of inflammatory cell accumulation than did MN10021 (75–95% versus 70% for MN10021); however, the doses of dexamethasone, acetaminophen, and diclofenac required to achieve maximal inhibition were 204, 6614, and 314 nmol, respectively. In contrast, maximal inhibition of inflammatory cell accumulation with MN10021 was achieved with only 1 nmol of peptide. Similarly, the IC_50_ for acetaminophen and diclofenac was 132 and 16 nmol respectively while that for MN10021 was only 0.1 nmol. The data presented here are from one experiment in which accumulation of monocytes/macrophages was quantitated 48 h after NaIO_4_ injection. Similar results were obtained on at least two other occasions with the same assay. Furthermore, similar results were also observed (data not shown) in two experiments in which neutrophil accumulation in the peritoneum was quantified 8 h after an i.p. injection of mice with NaIO_4_.

**Table 2:** Potency of MN10021 in Mouse Peritonitis Model of Inflammation.

Agent	Maximum Inhibition (%)	Dose Achieving Maximum Inhibition (µg/nmoles)	IC_50_ (µg/nmoles)
Dexamethasone	75	80/204	ND
Acetaminophen	95	1000/6614	20/132
Diclofenac	90	100/314	5/16
MN10021	70	4/1	0.4/0.1
MN20050	NE	NA	NA

ND  =  Not Determined.

NE  =  No Effect.

NA  =  Not Applicable.

### Effects of MN10021 in a Carrageenan Model of DIC

**Figure 1:**
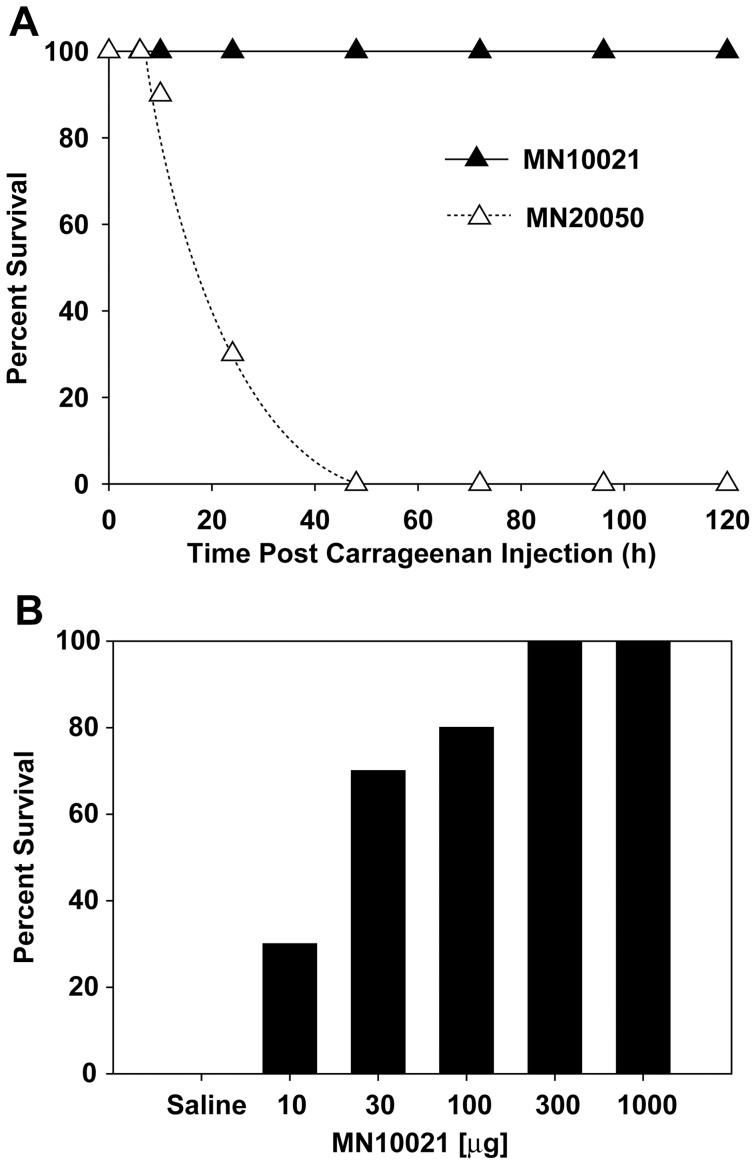
Effect of retroviral peptides on lethality associated with carrageenan-induced DIC in mice. A. Groups of 10 CD-1 mice (male, 6–8 weeks of age) were injected i.p. with 0.25 ml of PBS containing either 1 mg of either MN10021 or MN20050. Eighteen hours later the mice were injected i.p. with 1 ml of 0.45% carrageenan and survival was monitored over 48 h. The results shown are representative of >5 independent studies. B. Groups of 10 CD-1 mice (male, 6–8 weeks of age) were injected i.p. with the indicated doses of MN10021. Eighteen hours later the mice were injected i.p. with 1 ml of 0.45% carrageenan and survival was monitored over 48 h.

MN10021 displayed potent anti-inflammatory activity in the induced peritonitis model as described above. We also tested MN10021 in a carrageen-induced model of paw inflammation in mice and found it to be equally active (data not shown). We therefore sought to determine whether MN10021 might be active in a mouse model in which DIC is induced by the i.p. injection of carrageenan. As shown in Figure 1A, CD-1 mice injected i.p. with carrageenan rapidly become sick and die within 24–72 h. In contrast, mice previously injected i.p. with 1 mg (245 nmol) MN10021 maintain a normal physical appearance and readily survive to at least 120 h (longest time observed). We next tested various doses of MN10021 to obtain an estimate for an IC_50_ for protection by MN10021 in this model. As illustrated in Figure 1B, a dose of 30 µg (7 nmol) of MN10021, injected 18 h prior to the injection of carrageenan, protects approximately 50% of the mice. A number of other anti-inflammatory agents were also tested, with no success, for their ability to protect mice in this model of DIC (data not shown). These included neutralizing monoclonal antibody (Mab) to TNF-α, neutralizing Mab to IL-1β, dexamethasone, acetaminophen, diclofenac, indomethacin, naproxen, aminoguanidine, quinacrine, and staurosporine. Pentoxifylline, a drug used to treat peripheral tissue blood flow and reported to have some activity for inhibition of TNF-α release, had some protective activity but never achieved >50% protection.

### Effects of MN10021 on Cytokine Release in DIC Model

**Figure 2:**
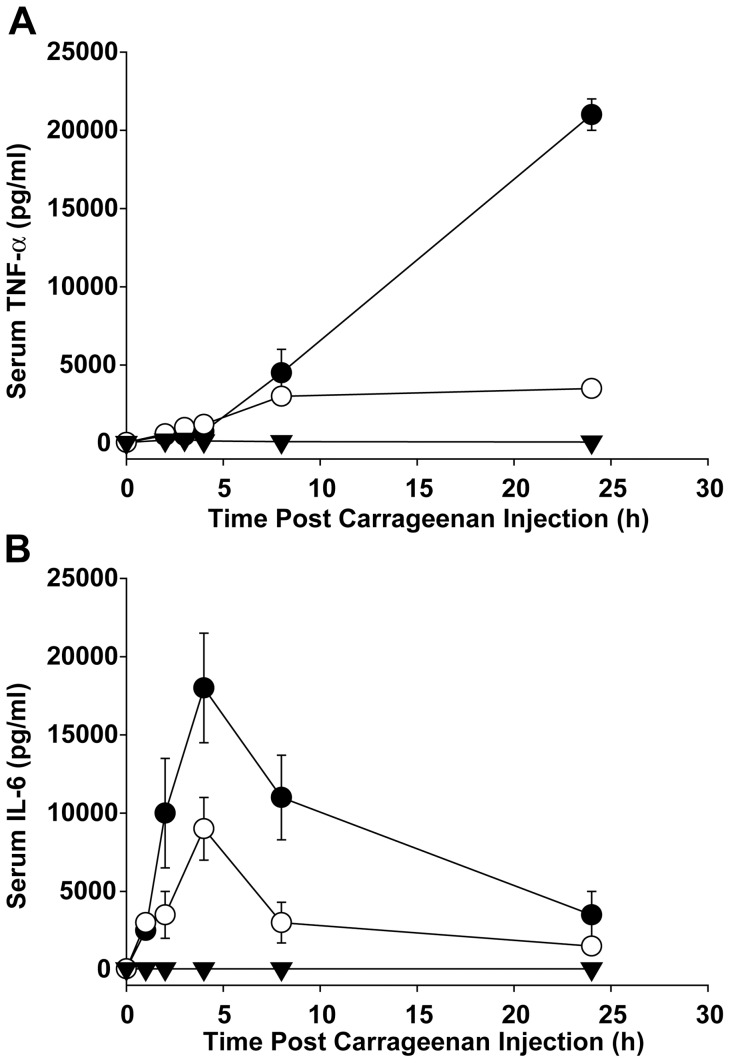
Inhibition of serum TNF-α and IL-6 levels in murine DIC model by MN10021. Each of 5 groups of 5 CD-1 mice (male, 6–8 weeks of age) were injected i.p. with 0.25 ml of either saline (solid inverted triangles), 1.0 mg (245 nmol) MN20050 in saline (closed circles), or 1.0 mg (245 nmol) MN10021 in saline (open circles). Eighteen hours later the MN20050 and MN10021 groups were injected i.p. with 1.0 ml of 0.45% carrageenan and the saline injected group received an additional 1.0 ml i.p. injection of saline. At each of the indicated times one group for each of the treatment conditions was euthanized, plasma was collected by cardiac puncture into heparinized syringes, and the plasma was stored frozen at −80°C. All samples were assayed for either (A) TNF-α or (B) IL-6 concentration using a solid-phase ELISA per the manufacturer's instructions. The data plotted are the mean values ± SE. Error bars were calculated for all values and if not visible are contained within the symbol.

In order to determine whether MN10021 affected cytokine release under conditions where it protected mice in the DIC model, mice were treated with MN10021 or saline and 18 h later injected with carrageenan. At 1-, 2-, 4-, 8- and 24-h post carrageenan injection, levels of TNF-α (Figure 2A) and IL-6 (Figure 2B) were determined from plasma samples by solid-phase ELISA. As Figure 2A shows, there was a steady increase in the level of TNF-α in the untreated, carrageenan-injected mice over 24 h. This increase was substantially (∼85%), but not completely blocked by pre-treating the mice with 1 mg of MN10021. In contrast to the data observed for TNF-α, in untreated mice injected with carrageenan, the increase in the plasma level of IL-6 peaked at 4 h and then declined ∼75% over the next 20 h (Figure 2B). Pretreatment with 1 mg (245 nmol) of MN10021 blocked about 50% of the peak response of IL-6. Although we have not previously observed MN10021 inhibition of IL-6 in human monocytes, inhibition of IL-6 by MN10021 in this mouse model might be the result of different species or the fact that additional cell types might be targeted by the MN10021.

### Effects of MN10021 on Hematological Parameters in DIC Model

**Figure 3:**
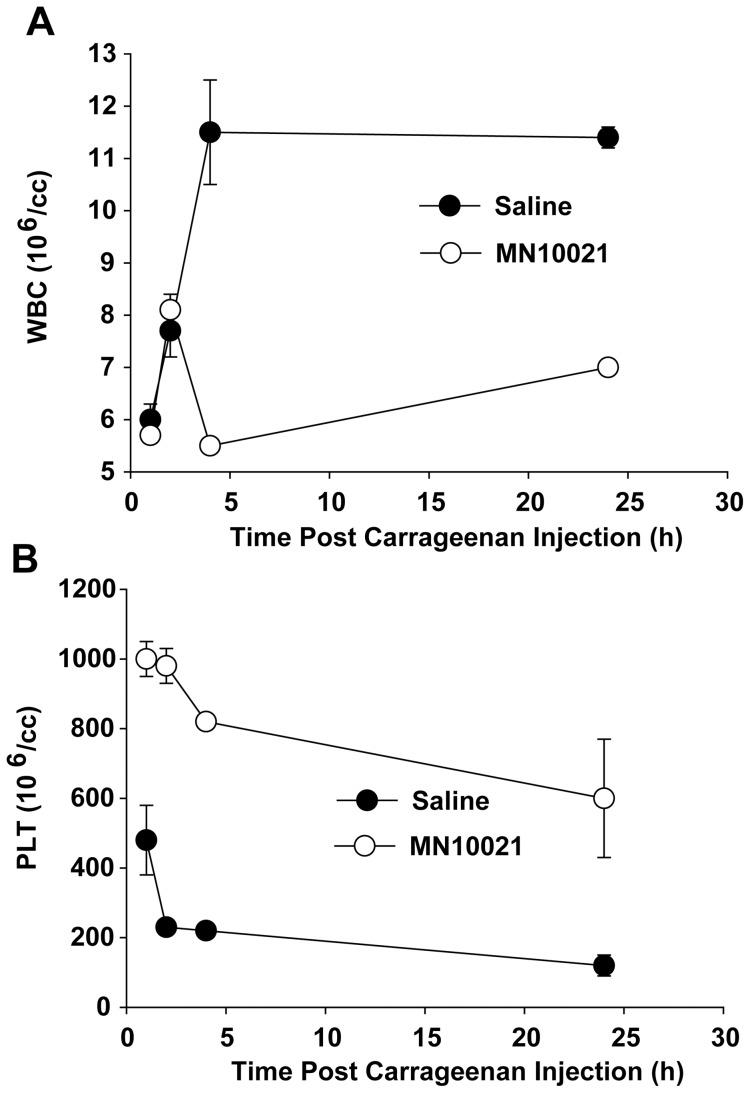
Effect of MN10021 on blood leukocyte and platelet counts in murine DIC model. Each of 4 groups of 5 CD-1 mice (male, 6–8 weeks of age) was injected i.p with 1.0 ml of either saline (closed circles) or 1.0 mg (245 nmol) MN10021 in saline (open circles). Eighteen hours later all mice were injected i.p. with 1.0 ml of 0.45% carrageenan. At each of the indicated times one group of 5 mice for each treatment was euthanized, an anti-coagulated blood sample obtained by cardiac puncture, and total white blood cell (WBC) (panel A) or platelet (PLT) (panel B) counts performed on an automated hematology analyzer. In both panels the dotted line represents the average value obtained from the blood samples of 10 untreated CD-1 mice. The data plotted are the mean values ± SE. Error bars were calculated for all values and if not visible are contained within the symbol.

We next examined several hematological parameters in the plasma of mice, untreated or treated with 245 nmol of MN10021, injected with a lethal dose of carrageenan. As shown in Figure 3A, mice injected with carrageenan experienced a sharp increase in white blood cell (WBC) counts at 2–4 h post injection followed by a gradual decrease back toward normal levels over the next 20–22 h. The mice injected with carrageenan also experienced a sharp (∼80%) decrease in platelet (PLT) levels during this same timeframe (Figure 3B), with the levels remaining decreased during the entire 24 h observation period. There was no observed effect on red blood cell (RBC) counts during the 24 h of observation (data not shown). Treatment with an i.p. injection of 245 nmol of MN10021 18 h prior to injection with carrageenan prevented much of the leukocytosis and inhibited a large percentage of the early decrease in PLT counts, although the PLT count of the MN10021-treated mice did decrease slowly over the 24 h period to approximately 50% of normal.

### Effects of MN10021 in Vascular Leak Assays

**Figure 4:**
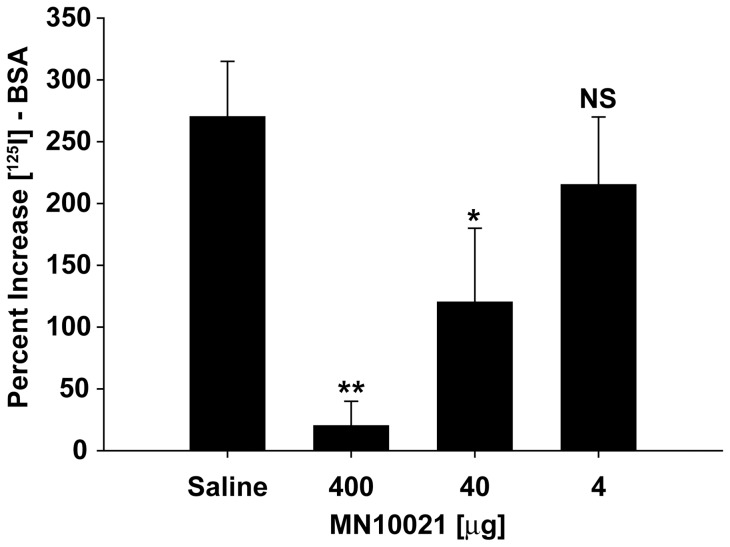
MN10021-mediated inhibition of vascular leak in murine DIC model. Two groups of 5 CD-1 mice (male, 6–8 weeks of age) were each injected i.p with 1.0 ml of either saline or 1.0 ml of the indicated dose of MN10021 in saline. Twelve hours later the mice were each injected i.v. with 0.25 ml of saline containing 10^6^ cpm of [^125^I]-BSA followed by an i.p. injection of 1.0 ml of either saline or 1.0% carrageenan. Twelve hours later the mice were euthanized, the lungs removed, and the lung associated radioactivity determined by gamma scintillation spectrophotometry. The increases in lung associated radioactivity was calculated as There were no differences in lung-associated radioactivity in mice (whether treated with MN10021 or not) injected i.p. with saline. The data plotted are the mean ± SE of the 5 measurements made. ** p<0.01; * p<0.05; NS  =  not significant.

Mice injected with carrageenan eventually presented with a frothy pink exudate from their respiratory tract and necropsy of mice which had died demonstrated hemorrhage in various internal organs, most noticeably the lungs. We thus examined the effect of MN10021 on vascular leak in the lungs of mice injected with carrageenan (Figure 4). Groups of 5 CD-1 mice were pretreated with the indicated doses of MN10021 or vehicle (saline) injected i.p. 12 h prior to i.p. injection of carrageenan or saline. Just prior to the injection of carrageenan or saline the mice were each injected i.v. with 0.25 ml of saline containing 10^6^ cpm of [^125^I]-BSA. Twelve hours after i.p. injection of carrageenan or saline the mice were euthanized, the lungs removed, and the percent increase in lung-associated radioactivity determined by comparing the radioactivity in the lungs of the carrageenan-injected animals with that in the lungs of the mice injected i.p. with saline. As Figure 4 shows, 12 h after injection there is an approximate 270% increase in radioactivity in the lungs of carrageenan-injected mice, suggesting an increase in vascular permeability. Injection with MN10021 inhibits this increase in vascular permeability in a dose-dependent manner, with >90% and >50% inhibition of the increase in vascular permeability in mice treated with the 400 and 40 µg doses of MN10021 respectively.

**Figure 5:**
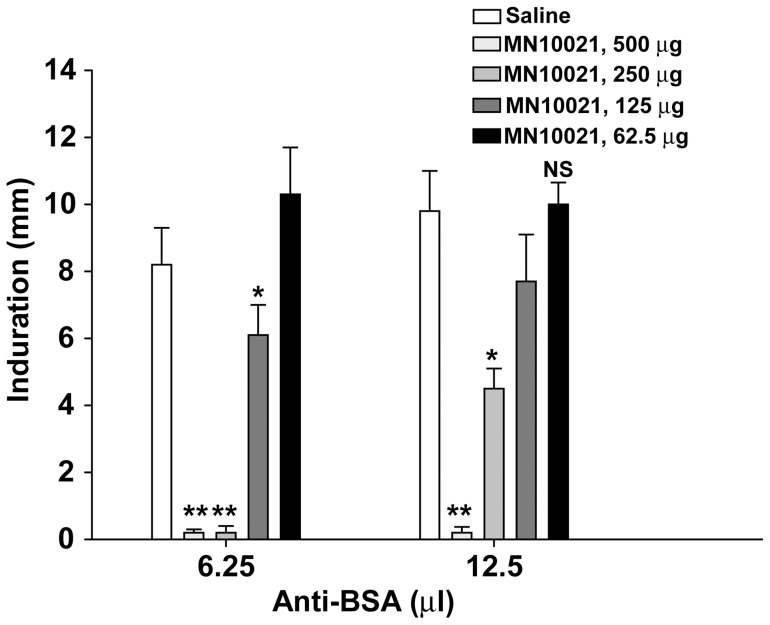
MN10021 inhibits dermal vascular leak in a reverse passive Arthus reaction. Groups of 5 Hartley strain guinea pigs (male; ∼250 g) were injected s.c. with 1.0 ml of either saline or the indicated dose of MN10021. Eighteen hours later the flanks of the animals were shaved and both 6.25 and 12.5 µl of rabbit anti-BSA antiserum injected i.d. at each of two sites, one on each flank. The animals were then injected i.v. with 1.0 ml of PBS containing BSA (1.0 mg/ml) and Evans Blue dye (2.0 mg/ml). Six hours later the animals were euthanized by barbiturate overdose, the skin on the back removed, and the diameter of the extravasation of the Evans Blue dye measured for each site on the ventral surface of the skin. The values represent the mean ± SE of the 10 measurements obtained for each of the treatment conditions. ** p<0.01; * p<0.05; NS  =  not significant.

In order to verify the inhibition of vascular leak by MN10021 in a second model, we tested MN10021 in a reverse passive Arthus reaction model in guinea pigs (Figure 5). Guinea pigs were injected s.c. with the indicated dose of MN10021 or vehicle (saline). Eighteen hours later both flanks of the animals were shaved and the indicated amounts of rabbit anti-BSA antiserum injected intradermally (i.d.) at two sites, one on each flank. The animals were then injected i.v. with 1 ml of PBS containing BSA (1 mg/ml) and Evans Blue dye (2 mg/ml). Six hours later the animals were euthanized by barbiturate overdose, the skin removed, and the diameter of the extravasation for each site measured on the ventral surface of the skin. As shown in Figure 5, either 250 or 500 µg of MN10021 completely blocked vascular leak in response to the lowest challenge dose (6.25 µl) of anti-BSA while only the highest dose of MN10021 completely blocked the higher challenge dose of 12.5 µl of anti-BSA. Thus, the ability of MN10021 to block vascular leak in this reverse passive Arthus reaction model appears to be dependent on both the dose of MN10021 and the dose of the challenge administered intradermally.

### Uptake of MN10021 by Human Monocytes and Endothelial Cells

**Figure 6:**
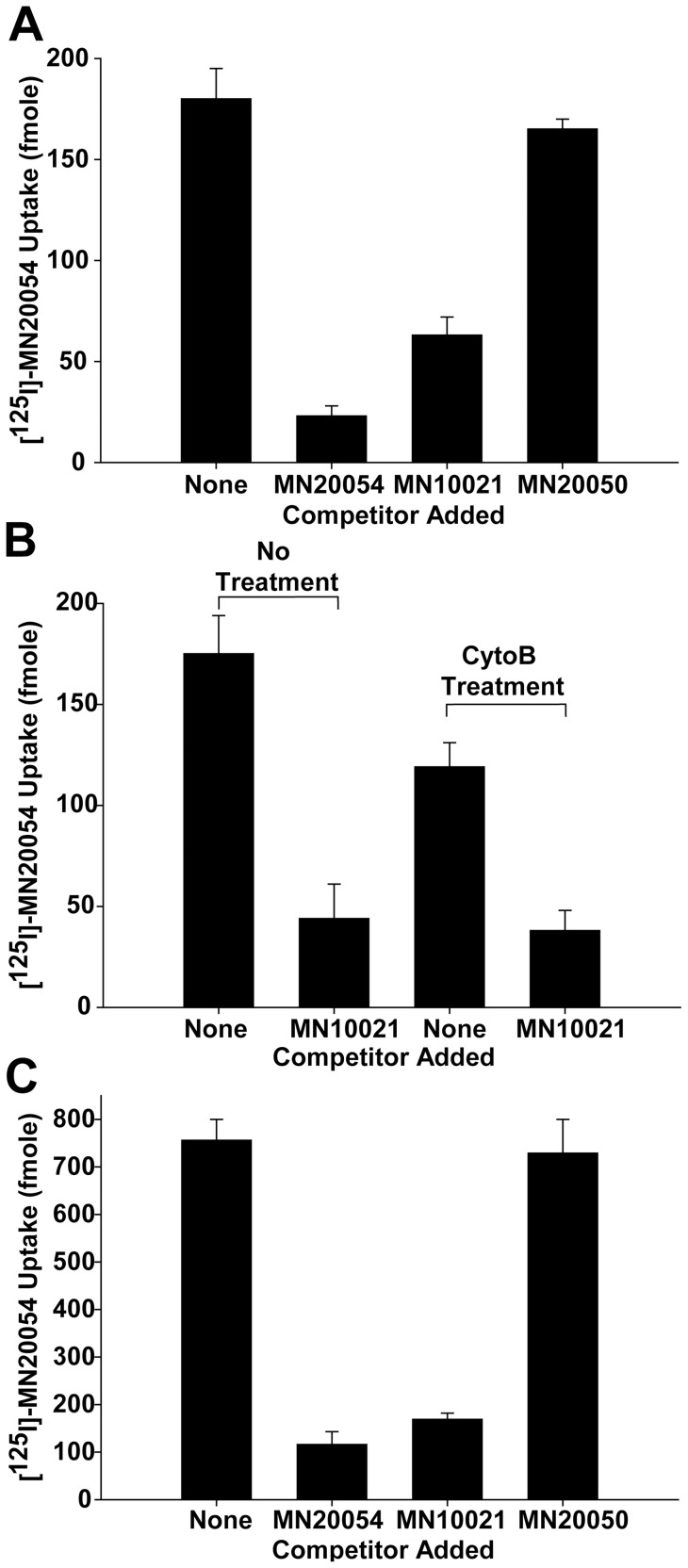
Monocyte and endothelial cell binding of MN10021 and MN20054. A. Human blood monocytes, isolated as described in Methods, were adjusted to 5×10^6^/ml in PBS (0.5% BSA, pH 7.5). Two-tenths ml of cell suspension was added to each of triplicate 12×75 mm polypropylene tubes and the tubes were incubated for 60 min at 37°C with 20 pmol of [^125^I]-MN20054 in the presence of either buffer alone, a 100-fold molar excess of MN20054, a 100-fold molar excess of MN10021, or a 100-fold molar excess of MN20050. The contents of each tube were then layered onto 0.8 ml of 10% sucrose in Eppendorf tubes, centrifuged, and the cell-associated radioactivity determined by gamma scintillation spectrophotometry of the cell pellet. B. Binding/uptake of [^125^I]-MN20054 by human monocytes was performed as described for panel A with the following exceptions: (1) half the cells were pretreated for 30 min at 37°C with cytochalasin B to inhibit endocytosis and (2) competition was performed only with a 100-fold molar excess of MN10021. C. HUVEC were grown to confluence in 12-well tissue culture plates. To each of triplicate wells was added 20 pmol of [^125^I]-MN20054 in the presence of either buffer alone, a 100-fold molar excess of MN20054, a 100-fold molar excess of MN10021, or a 100-fold molar excess of MN20050. The plates were incubated for 60 min at 37°C with gentle rocking, the wells aspirated and quickly washed 3X with 2.0 ml each of ice-cold PBS/BSA and 1.0 ml of 1.0 N NaOH added to each well to solubilize the cells. Nine-tenths ml of solubilized cells was then removed from each well and cell-associated radioactivity determined by gamma scintillation spectrophotometry.

The ability of MN10021 to inhibit vascular leak raised the question of whether it might be affecting not only blood leukocytes but also having a direct effect on vascular endothelial cells. We first sought to determine whether MN10021 bound to or was taken up by human blood monocytes or endothelial cells. An analog of MN10021, MN20054, was synthesized in which the amino acid Phe at the 11 position was replaced with a Tyr to allow the peptide to be labeled with [^125^I]. Binding isotherms using [^125^I]-MN20054 and purified, blood-derived human monocytes indicated that [^125^I]-MN20054 appeared to bind in a specific, saturable manner (data not shown). However, because of insolubility of MN10021 at lower temperatures, these studies were performed at 37°C which raised the issue of whether binding or uptake was measured. Using a dose of [^125^I]-MN20054 that gave maximal cell-associated activity, we examined the association of [^125^I]-MN20054 with human monocytes in the presence or absence of a 100-fold molar excess of either unlabeled MN20054 or unlabeled MN10021 (Figure 6A). As the data indicate, the binding or uptake of [^125^I]-MN20054 is inhibited by ∼85% by unlabeled MN20054 and by ∼65% by unlabeled MN10021. The unlabeled control peptide MN20050 (reverse sequence) inhibited cell association of [^125^I]-MN20054 by <10%. In order to determine whether the cell-associated radioactivity represented binding or uptake, the same experiment was performed in the presence of cytochalasin B to inhibit uptake. As shown in Figure 6B, in the presence of cytochalasin B there is a ∼40% reduction in the amount of [^125^I]-MN20054 associated with the monocytes that is not inhibited by MN10021. Thus a significant portion of the association of MN10021 with human blood monocytes appears to be binding, rather than uptake and internalization.

We next sought to determine if MN10021 also associated with human vascular endothelial cells. Using the same methodology as used for monocytes, [^125^I]-MN20054 was incubated with HUVEC in the presence or absence of a 100-fold excess of unlabeled MN20054 or MN10021. As shown in Figure 6C, there is demonstrable association of [^125^I]-MN20054 with HUVEC which is substantially inhibited (84 and 77%) by unlabeled MN20054 and MN10021, respectively, while there was no significant inhibition by the control peptide, MN20050. Thus MN10021 also binds to or is taken up by human vascular cells.

**Figure 7:**
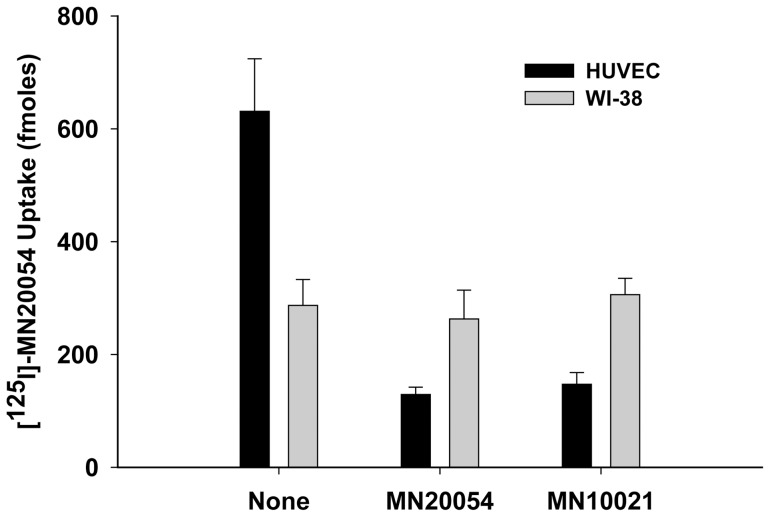
Binding of [^125^I] – MN20054 to Fibroblasts. HUVEC and WI-38 fibroblasts (CCL-75™; ATCC) were grown to confluence in 12-well tissue culture plates. To each of triplicate wells was added 20 pmol of [^125^I]-MN20054 in the presence of either buffer alone, a 100-fold molar excess of MN20054, or a 100-fold molar excess of MN10021. The plates were incubated for 60 min at 37°C with gentle rocking, the wells aspirated and quickly washed 3X with 2.0 ml each of ice-cold PBS/BSA and 1.0 ml of 1.0 N NaOH added to each well to solubilize the cells. Nine-tenths ml of solubilized cells was then removed from each well and cell-associated radioactivity determined by gamma scintillation spectrophotometry.

Although we did not specifically examine binding of [^125^I]-MN20054 to human lymphocytes, we have reported the biological effects of MN10021 on human T- and B-lymphocyte responses and NK cell responses as well as on a murine T-cells [10], [11], [12], [15] suggesting that the peptides either bind to or are taken up by these non-adherent cells. In order to determine if [^125^I]-MN20054 binds to human fibroblasts we utilized the human fibroblast cell line WI-38. As shown in Figure 7, while the binding/uptake of [^125^I]-MN20054 by HUVEC was inhibited ∼75% by a 100-fold excess of either unlabeled MN20054 or MN10021, the binding/uptake of [^125^I]-MN20054 by the WI-38 cells was unaffected by the 100-fold excess of either unlabeled peptide, suggesting that all of the binding/uptake is non-specific. This result, although with human-derived cells, would seem to support previous studies demonstrating a lack of effect by this peptide sequence on either murine fibroblast proliferation [5], [20] or protein synthesis [21].

### Effects of Oral MN10021 on Blood Pressure in SHR Rats

**Figure 8:**
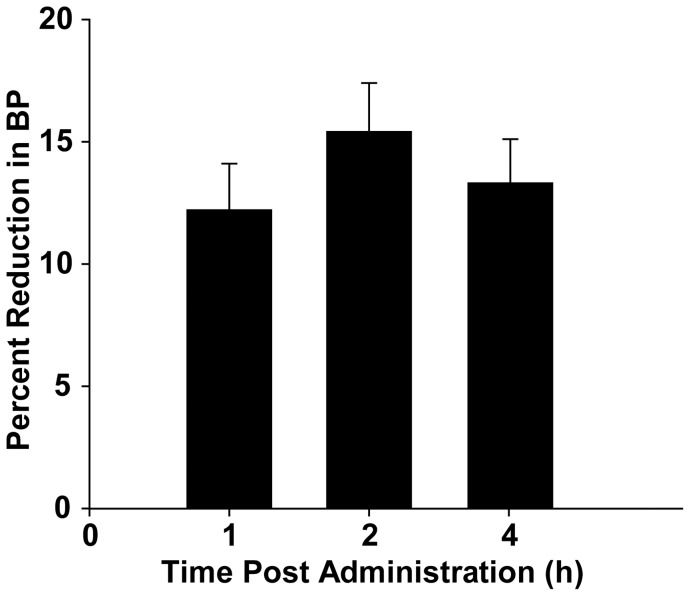
MN10021 lowers blood pressure in SHR rats. Three SHR rats were orally-dosed (by gavage) with MN10021 (100 mg/kg) prepared in 2% Tween 80/distilled water. Systolic blood pressure (BP) measurements were made using a tail-cuff manometer at 0, 1, 2, and 4 h. L-methyldopa (100 mg/kg) was used as a positive control. The percent reduction in BP is calculated based on the 0 h value and is expressed as a mean ± SE. The experiment shown is representative of two separate experiments.

As part of a general pharmacological screen, MN10021 was tested in a SHR rat model for measuring anti-hypertensive activity of compounds. Groups of three SHR rats were orally-dosed (by gavage) with MN10021 (30 or 100 mg/kg). Blood pressure measurements were made at 1, 2 and 4 h using a tail-cuff manometer. As shown in Figure 8, 100 mg/kg (24 µmol/kg) MN10021, dosed orally, causes a rapid 12% decrease in blood pressure within 1 h of oral dosing and reaches a maximal decrease of 15% within 2 h. At 4 h, the maximal time observed, there was still a 13% decrease in blood pressure from baseline levels. At the 30 mg/kg dose of MN10021 there was detectable but minimal (3–4%) decreases in blood pressure at the three time points measured (data not shown). The positive control L-methyldopa, dosed orally at 100 mg/kg (474 µmol/kg), lowered blood pressure by 16, 23, and 28% at 1, 2, and 4 h, respectively (data not shown).

### Stimulation of inducible nitric oxide synthase (iNOS; NOS2) in HUVEC by MN10021

**Figure 9:**
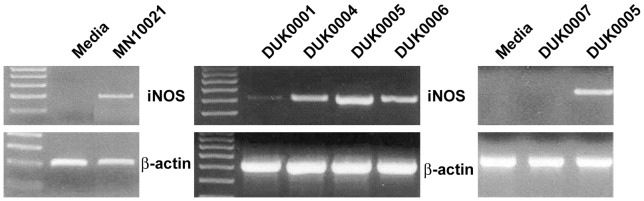
Induction by MN10021 and analogs of mRNA for iNOS in HUVEC. A. HUVEC were grown to 80–90% confluence in 12-well tissue culture plates using defined media provided by Cambrex and supplemented with 10% FBS. The cells were allowed to become quiescent by incubating them overnight in the basal media (without growth factors) supplied by Cambrex and supplemented with 1% FBS. Triplicate wells were incubated with either media alone or media containing 50 µM MN10021 for 3 h at 37°C, the wells washed with warm PBS, and the cells lysed. Total RNA was isolated, cDNA prepared, and RT-PCR performed as described in Materials & Methods. Primers for human iNOS and β-actin were obtained from R & D systems. B. HUVEC were prepared as described under Methods, grown to 80–90% confluence in 12-well tissue culture plates, and incubated overnight in basal medium (no growth factors) containing 1% FBS in order to make them quiescent. Triplicate wells were incubated for 3 h at 37°C with 50 µM of one of four different 8-amino acid long analogs of MN10021. DUK0001: NH_2_-GLDLLFLK-COOH; DUK0004: acetyl-GLDLLFLK-acetyl; DUK0005: acetyl-GLDLLFLK-NH_2_; DUK0006: NH_2_-GLDLLFLK-NH_2_. The wells were washed with warm PBS, and the cells lysed. Total RNA was isolated, cDNA prepared, and RT-PCR performed as described in Materials & Methods. Primers for human iNOS and β-actin were obtained from R & D systems. C. HUVEC were prepared and tested as described in panel A. The sequence of DUK0007 is acetyl-GLDLLYLK-NH_2_ which differs from that of DUK0005 in that it has a Tyr at position 6 in place of a Phe.

One mechanism by which a compound might lower blood pressure is to induce the release of nitric oxide (NO) into the blood. Since MN10021 had been found to bind to or be taken up by endothelial cells and since endothelial cells are a known source of NO, we examined the effect of MN10021 on the induction of mRNA for iNOS in HUVEC. HUVEC were incubated for 3 h with MN10021 and the mRNA for iNOS examined using RT-PCR. As shown in Figure 9A, MN10021 induces mRNA for iNOS within 3 h.

### Identification of Potential Active Site in MN10021

MN10021 dosed orally caused a rapid (1 h) drop in blood pressure in SHR rats (Figure 8). It seemed unlikely that MN10021, a dimerized octadecapeptide, would enter the bloodstream intact within this short period of time. Moreover, we presently have no evidence that MN10021 is absorbed rapidly through the oral or gastric mucosa and in the study of Figure 8, the MN10021 was delivered by gavage, bypassing the oral mucosa. We therefore next considered what fragment(s) of MN10021 might survive degradation in the gastrointestinal tract and be absorbed into the bloodstream and determined that the portion of MN10021 flanked by Arg-Arg on the amino terminus and by Lys on the carboxy terminus would be most likely to survive. The first peptide fragment we tested was an 8-amino acid peptide, GLDLLFLK, termed DUK0001. Incubation of DUK0001 with HUVEC for 3 h, as was done with MN10021, resulted in a weak induction of mRNA for iNOS but increasing the time of incubation to 5 h (data not shown) or making modifications to increase the solubility or stability of the peptide resulted in a much stronger induction (Figure 9A). These modifications included acetylation of both the amino- and carboxy-termini (DUK0004), acetylation of the amino-terminus and amidation of the carboxy-terminus (DUK0005), and amidation of both the amino- and carboxy-termini (DUK0006). As shown in Figure 9B, although DUK0004 is the least soluble of these three peptides and DUK0006 is the most soluble, they are comparable in their abilities to induce iNOS in HUVEC. DUK0005, which is intermediate of the three peptides in solubility, is the most effective in inducing iNOS.

Neither MN10021 nor DUK0005 affected the expression of mRNA for endothelial nitric oxide synthase (eNOS) which was constitutively expressed in HUVEC (data not shown).

### Activity of DUK0005 is Sequence Specific

In order to verify that the iNOS-inducing activity of DUK0005 was related to the specific amino acid sequence, an analog (DUK0007) was prepared and tested in which the Phe at the 6 position was replaced by a Tyr. This change results in the replacement of one hydrophobic amino acid with another hydrophobic amino acid but one that contains an additional hydroxyl group. As shown in Figure 9C, DUK0007, which is structurally-identical to DUK0005 except for the replacement of the Phe by the Tyr, does not induce iNOS in treated HUVEC. Interestingly, when DUK007 is combined 1∶1 with DUK005, it inhibits the induction of iNOS by DUK005 (data not shown) suggesting that it might bind or be taken up by the cells but acts as a competitive antagonist. However, we have not ruled out that this might simply be due to peptide-peptide interactions.

### MN10021 and DUK0005 Protect HUVEC from Apoptosis

**Figure 10:**
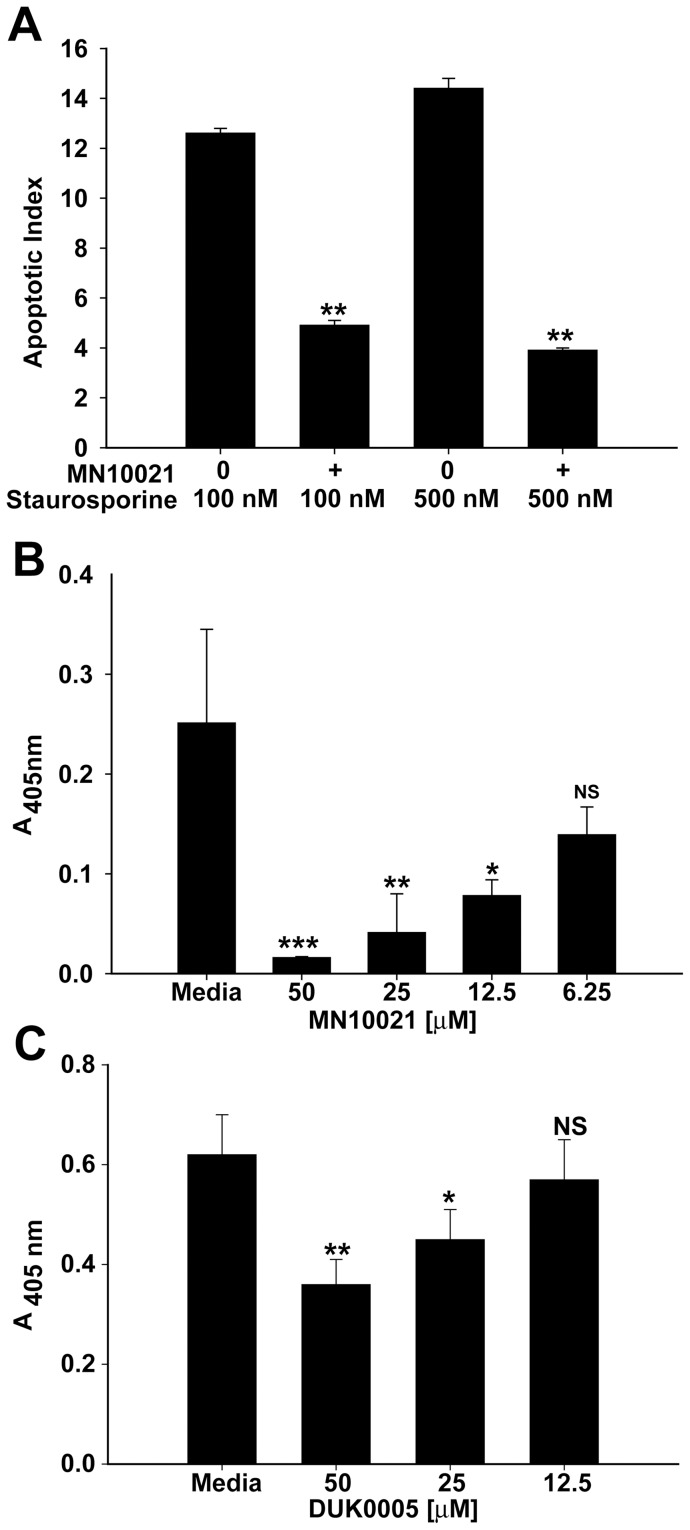
MN10021 and DUK0005 inhibit apoptosis and necrosis in HUVEC. A. HUVEC were grown to confluence in 96-well tissue culture plates. Media was removed from the wells and to each of quadruplicate wells was added 200 µl of either fresh media (untreated) or fresh media containing 50 µM MN10021 (treated) and the cells incubated 3 h at 37°C. To quadruplicate wells of both untreated and treated cells were added 20 µl of media or 20 µl of media containing either 100 or 500 nM staurosporine. The plates were incubated overnight at 37°C and the cells processed for measurement of apoptosis using the Cell Death Detection assay (Roche Diagnostics) per the manufacturer's instructions. The apoptotic index was calculated as described in Methods. B. HUVEC were grown to confluence in 96-well tissue culture plates. Media was removed from the wells and to each of quadruplicate wells was added 200 µl of either fresh media (untreated) or fresh media containing 50, 25, 12.5, or 6.25 µM MN10021 (treated) and the cells incubated 3 h at 37°C. To quadruplicate wells of both untreated and treated cells were added 20 µl of media containing 100 ng/ml human TNF-α (recombinant; R & D Systems). The plates were incubated overnight at 37°C and the supernatants removed and processed for measurement of necrosis using the Cell Death Detection assay (Roche Diagnostics) per the manufacturer's instructions. Absorbance values for the supernatants of untreated cells (no TNF-α) were negligible. C. HUVEC were grown to confluence in 96-well tissue culture plates. Media was removed from the wells and to each of quadruplicate wells was added 200 µl of either fresh media (untreated) or fresh media containing 50, 25, 12.5 µM DUK0005 (treated) and the cells incubated 3 h at 37°C. To quadruplicate wells of both untreated and treated cells were added 20 µl of media or 20 µl of media containing 100 nM staurosporine. The plates were incubated overnight at 37°C and the cells processed for measurement of apoptosis using the Cell Death Detection assay (Roche Diagnostics) per the manufacturer's instructions. Absorbance values for untreated cells (no staurosporine) were negligible. *** p<0.001; ** p<0.01; * p<0.05.

Although NO associated with inflammatory reactions is often considered to be pro-apoptotic, there are a number of studies indicating that low or physiological levels of NO can prevent apoptosis and can be vasoprotective in various *in vivo* situations. We, therefore, examined the ability of MN10021 and DUK0005 to protect treated HUVEC against staurosporine-induced apoptosis. As shown in Figure 10A, MN10021 added to HUVEC 3 h before the addition of staurosporine (100 or 500 nM) resulted in a significant protection against staurosporine-induced apoptosis measured at 24 h after staurosporine addition. Attempts to measure apoptosis of HUVEC treated with TNF-α were unsuccessful in that the TNF-α caused a rapid necrosis of the HUVEC. However, even in this situation, pretreatment of the HUVEC with MN10021 for 3 h prior to addition of the TNF-α resulted in a significant protection against the necrosis-inducing effects of the TNF-α (Figure 10B). Treatment of HUVEC with DUK0005 for 3 h prior to addition of staurosporine also resulted in a dose-dependent inhibition of apoptosis (Figure 10C) although the maximal protection afforded by DUK0005 (∼40%) was less than the maximal protection afforded by MN10021 (∼60%) as shown in Figure 10A. An explanation for this difference in protection has not yet been determined.

### Does NO synthesis correlate with vasoprotection

**Figure 11:**
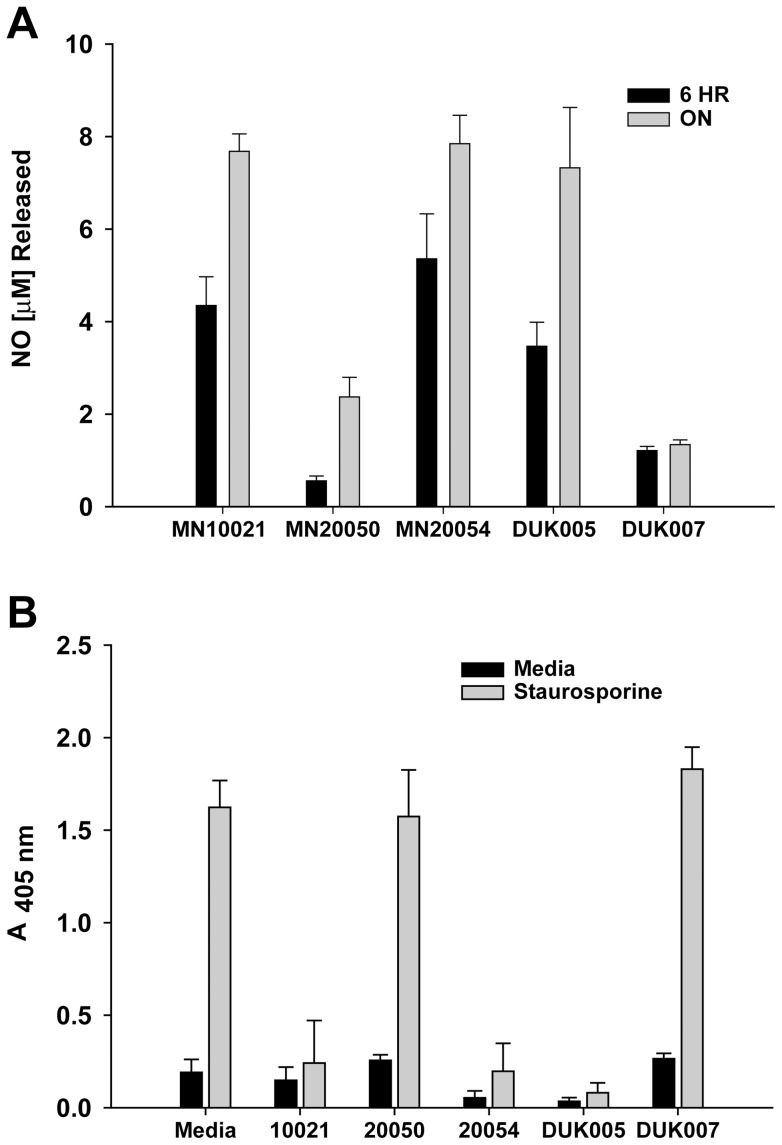
Inhibition of apoptosis in HUVEC correlates with production of NO by retroviral-derived peptides. A. HUVEC were grown to 70–80% confluence in 96-well tissue culture plates. Media was removed from the wells and to each of quadruplicate wells was added 200 µl of either fresh media (untreated) or fresh media containing 50 µM of the indicated peptides (treated) and the cells incubated ON at 37°C. Supernatant was removed from each well as assayed for total NO by measurement of nitrite with a Colorimetric Nitric Oxide Assay Kit (Oxford Biomedical Research; Oxford, MI) which employs nitrate reductase to convert nitrate to nitrite. B. HUVEC were grown to confluence in 96-well tissue culture plates. Media was removed from the wells and to each of quadruplicate wells was added 200 µl of either fresh media (untreated) or fresh media containing 50 µM of the indicated peptide (treated) and the cells incubated 3 h at 37°C. To quadruplicate wells of both untreated and treated cells were added 20 µl of media or 20 µl of media containing 500 nM staurosporine. The plates were incubated overnight at 37°C and the cells processed for measurement of apoptosis using the Cell Death Detection Assay (Roche Diagnostics) per the manufacturer's instructions.

MN10021 and DUK005 both induced iNOS mRNA and both protected against staurosporine induced apoptosis, but we had not actually demonstrated NO release by these peptides. In order to verify that the induction of iNOS by these peptides is accompanied by the release of NO, we measured the release of nitrate into cell culture supernatants of HUVEC that had been incubated with peptides. As shown in Figure 11A, peptides MN10021, MN20054, and DUK005, which either induced iNOS when incubated with HUVEC (MN10021 and DUK005) or competed for binding with MN10021 (MN20054), all induced nitrate release (as measured by nitrite assay) from HUVEC incubated for either 6 h or ON with 50 µM peptide. The control peptide MN20050 (reverse sequence), which does not compete for [^125^I]-MN20054 binding and has no significant biological activity, and the DUK007 peptide, which did not induce iNOS after 3 h of incubation with HUVEC, both induced a small amount (≤25% of MN10021) of nitrate release after 6 h and ON incubations. Whether this nitrate was the result of iNOS induction is unknown. We, therefore, examined the ability of MN10021, MN20054 and DUK0005 to protect treated HUVEC against staurosporine-induced apoptosis. As shown in Figure 11B, addition of MN10021, MN20054, or DUK005 to HUVEC 3 h before the addition of staurosporine (500 nM) resulted in a significant protection against staurosporine-induced apoptosis measured at 18 h after staurosporine addition. The addition of either MN20050 or DUK007, neither of which produced significant NO, had no protective effective against staurosporine-induced apoptosis.

### Protective effect of peptides is not NO-dependent

**Figure 12:**
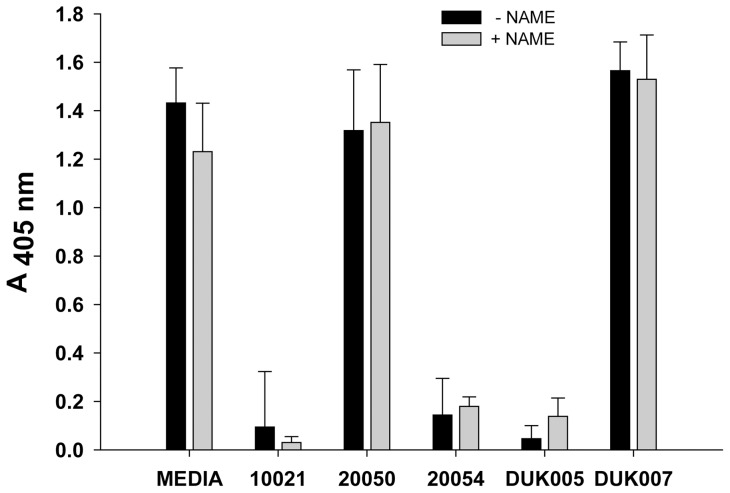
Pretreatment with L-NAME does not affect peptide inhibition of apoptosis. HUVEC were grown to 70–80% confluence in a 96-well tissue culture plate. Media was removed from the wells and to each of quadruplicate wells was added 200 µl of either fresh media (untreated) or fresh media containing 2 mM L-NAME and the cells incubated 5 h at 37°C. To each well was then added the indicated peptide to achieve a concentration of 50 µM and the cells incubated an additional 3 h at 37°C before addition of staurosporine to a final concentration of 500 nM. The plate was incubated ON at 37°C and the cells processed for measurement of apoptosis using the Cell Death Detection Assay (Roche Diagnostics) per the manufacturer's instructions.

In order to confirm that the protective effects of MN10021, MN20054, and DUK005 against staurosporine-induced apoptosis were the result of NO-induction, we pretreated HUVEC with L-NAME and then peptides prior to addition of staurosporine. As shown in Figure 12, pretreatment with the inhibitor of iNOS induction had no effect on the protective effects of MN10021, MN20054 or DUK005. Under these same conditions the release of NO into the media by the peptides was blocked by the pretreatment with L-NAME (data not shown).

## Discussion

Over two decades ago we first identified that human tumors produced anti-inflammatory proteins that were antigenically-related to the retroviral transmembrane (TM) protein p15E [1] and that p15E-related antigens were expressed not only in human tumors but in mitogen-transformed blood lymphocytes as well [22]. We subsequently showed that p15E itself had potent anti-inflammatory activity in a murine model of induced peritonitis [3] and that p15E contained a sequence of 26 amino acids that is highly-conserved amongst all the TM proteins of retroviruses, both in humans and other species [4]. Studies using a monomeric heptadecapeptide (CKS-17), corresponding to the first 17 amino acids of this highly-conserved domain (the “immunosuppressive domain”) and coupled to a carrier protein, have demonstrated both *in vitro* and *in vivo* activities, including inhibition of a wide variety of immunological functions [5], [6], [7], [8], [9], [10], [11], [12], [13].

MN10021, the homodimeric octadecapeptide, corresponding to the 17-amino acid sequence of CKS-17 with an additional (naturally-occurring) carboxy-terminal cysteine though which homodimers are readily formed, displays all of the biological activities of carrier-coupled CKS-17. It has been used to examine the molecular basis of the *in vitro* activities of these highly-conserved retroviral sequences [15], [16], [17], [18]. In these studies we now report for the first time a comprehensive pharmacological profile of MN10021 on human peripheral blood-derived leukocytes. The *in vitro* anti-inflammatory profile exhibited by MN10021 in Table 1 would suggest that it might have *in vivo* anti-inflammatory activity as well. Indeed, as shown in Table 2, MN10021 is a potent anti-inflammatory agent in a murine model of induced peritonitis. On a molar basis it is several orders of magnitude more potent than known anti-inflammatory compounds such as dexamethasone, acetaminophen or diclofenac.

One of the more interesting observations to emerge from these studies were the data shown in Figure 1 demonstrating the ability of MN10021 to protect mice from a lethal dose of carrageenan in a model that closely mimics disseminated intravascular coagulation (DIC). MN10021 dramatically inhibited (>80%) the large increase in serum TNF-α levels (>20 ng/ml) associated with a lethal outcome in this model (Figure 2A). The observation that neutralizing monoclonal antibodies to TNF-α did not protect in this model suggested that additional factors were involved. MN10021 also inhibited, but to a lesser extent (∼50%), the transient increase in IL-6 levels which also occurred in this model. The ability of MN10021 to inhibit both the leukocytosis and the thrombocytopenia in mice injected with a lethal dose of carrageenan (Figure 3A, 3B) suggests that its protective effect may be reflective of a broad array of activities, including its ability to inhibit *in vitro* both ADP- and epinephrine-induced human platelet aggregation (data not shown).

In both the carrageenan-induced DIC model and the guinea pig reverse passive Arthus reaction model, MN10021 afforded significant protection against vascular leak (Figures 4 and 5). Although the exact mechanism of this protection remains to be determined, these observations led us to examine vascular cells as potential targets for MN10021 and related sequences. Using a radiolabeled analog of MN10021, we demonstrated that MN10021 binds to or is taken up in a specific manner not only by human blood monocytes but also by human endothelial cells while the observed binding to human fibroblasts appears to be non-specific. This latter result is in agreement with previous results indicating a lack of effects by this peptide sequence on murine fibroblasts [5], [20], [21]. The ability of MN10021 to prevent vascular leak and to bind to vascular endothelial cells, coupled with the surprising observation that orally-dosed MN10021 lowers blood pressure in SHR rats within 1 h of oral-dosing, suggested that the anti-inflammatory activity of MN10021 might be related to its ability to maintain vascular integrity under inflammatory conditions. Since leukocytes must extravasate from the blood to sites of inflammation, a compound which protects the vascular endothelium might also be expected to be anti-inflammatory.

The ability of MN10021 to significantly lower blood pressure within 1 h of oral dosing suggested a potent vasodilatory effect. One of the most potent vasodilators known is nitric oxide (NO), first described as EDRF (endothelial-derived relaxation factor). Our studies show that a 3 h treatment of HUVEC with MN10021 upregulates mRNA for iNOS, the inducible enzyme responsible for the synthesis of NO in a variety of cell types such as monocytes/macrophages, endothelial cells, and vascular smooth muscle cells. These results led us to test the hypothesis that the ability of MN10021 to lower blood pressure in SHR rats is the result of induced synthesis of NO. Our hypothesis was supported by previous work by others. Although supra-normal levels of NO, often found in inflammatory lesions, are generally considered to be deleterious, low or physiological levels of NO are vasoprotective [23], [24], [25]. For example, physiological levels of NO have been reported to attenuate: IL-2-induced lung injury in rats [26]; phorbol ester-induced microvascular permeability in dogs [27] and hydrogen peroxide-induced vascular permeability in rabbits [28]; leukocyte adhesion and vascular leak after myocardial ischemia [29]; platelet aggregation and adherence to sites of injury on endothelial cells [30], [31], [32], [33], [34], [35]; upregulation of cell adhesion molecules (CAM) on endothelial cells [36], [37], [38], [39]; and vascular smooth muscle cell proliferation and migration [40], [41], [42]. Furthermore, NO inhibits leukocyte migration in a rat carrageenan-induced pleurisy model [43]. A subsequent study by different investigators, utilizing inhibitors of NOS in a rat carrageenan-induced pleurisy model, demonstrated that local production of NO was protective by virtue of its ability to regulate the release of inflammatory mediators and that NOS inhibitors had differential anti-inflammatory effects depending on their route of administration [44]. Although we have detected s.c.-injected MN10021 in the blood of mice within 15 min (data not shown) we have no evidence that MN10021 is readily absorbed through oral or gastric mucosal surfaces. We, therefore, began to investigate whether analogs of MN10021, representing small fragments which might survive the harsh environment of the stomach, could also induce mRNA for iNOS. We identified an 8-amino acid peptide, DUK0005, which is as effective as MN10021 in inducing iNOS mRNA in HUVEC and HUASMC. Of particular interest, other investigators previously reported that a similar peptide corresponding to the central six amino acids of DUK0005, LDLLFL, inhibited *in vitro* human monocyte chemotactic responses and ligand (CD3) and interleukin-2 (IL-2)-induced lymphocyte proliferation [45], [46]. However, these same investigators did not find this hexapeptide to be anti-inflammatory in either mice or guinea pigs although they did present several possible explanations [47].

Additional investigators have also identified p15E-derived peptides, partially homologous to MN10021, which have immunomodulator activities. A recent study reported on studies utilizing a peptide (analog 3; LDLLFLKEGGL) which differs from our DUK0005 in that it is missing the Gly on the amino terminus and it contains the additional four amino acids (EGGL) on the carboxy terminus [48].

In addition to the above-listed protective effects which have been reported for NO, physiological levels of NO have been shown to inhibit apoptosis in a number of cell types, including leukocytes [49], [50], [51], [52], hepatocytes [53], [54], [55], [56], [57], trophoblasts [58], [59] and endothelial cells [60], [61], [62], [63], [64], [65]. We therefore investigated whether MN10021 or DUK0005 could prevent apoptosis induced in HUVEC by staurosporine, a potent inhibitor of protein kinase C. Indeed, both MN10021 and DUK0005 protect against staurosporine-induced apoptosis in HUVEC. Although the percent inhibition of apoptosis was ∼60% and ∼40% for MN10021 and DUK0005, respectively, staurosporine is an extremely potent inducer of apoptosis with a very steep dose curve after a threshold dose concentration is attained. Thus, it is uncertain whether staurosporine-induced apoptosis is representative of what might occur with vascular cells under inflammatory conditions. In contrast, while TNF-α, in the absence of added cycloheximide, did not readily induce apoptosis, it did cause necrosis of HUVEC as measured by the release into the supernatant of histone/DNA fragment complexes. MN10021 was able to block almost completely, in a dose-dependent manner, this TNF-α-mediated necrosis of HUVEC. Of interest, in preliminary *in vivo* studies with MN10021 and DUK005, intraperitoneal injection of 1 µg of either peptide was able to inhibit the vascular leak in the liver of mice injected intravenously with dengue virus.

We have now confirmed the release of NO with our peptides (MN10021 and DUK005; Figure 11A) which induced iNOS mRNA as well as with MN20054 which competes for MN10021 binding. We have also demonstrated that these same peptides protect against staurosporine-induced apoptosis (Figure 11B) while our peptides (MN20050 and DUK007) that produced reduced amounts of NO (Figure 11A) had no protective effect against staurosporine. However, when HUVEC were pretreated with the inhibitor of iNOS, L-NAME, the protective effects of MN10021, MN20050, and DUK005 were not affected (Figure 12) even though NO release was blocked. Thus, the vasoprotective effects of these retroviral-derived peptide sequences appear to be independent of their ability to produce NO.

We and others have previously reported association of retroviral p15E-related proteins with a variety of human neoplasms and certain normal tissues [1], [22], [66], [67], [68], [69]. In addition, human endogenous retroviruses (HERV) encoding for membrane proteins which contain the highly-conserved region from which MN10021 is derived, have also been identified in both normal and malignant human tissues [70]–[83]. A protein or peptide with both anti-inflammatory and vasoprotective properties might be expected to confer a selective advantage to malignant cells expressing it.

Similarly, such a protein or peptide might be useful to cells such as trophoblasts that need to both survive in a hostile environment and to increase their blood supply and grow and expand. Indeed, in ongoing studies we have now demonstrated that peptides derived from the MN10021-homologous regions of ERV3 [84] and syncytin [79], two human endogenous retroviruses expressed in placenta, induce both iNOS and VEGF in vascular cells (Cianciolo and Pizzo; manuscript in preparation). Of particular interest, studies have reported that the levels of syncytin in placentas of preeclamptic women are dramatically decreased compared to women with normal pregnancies [80], [81], [83].

Such anti-inflammatory/vasoprotective proteins or peptides could also play a natural role as immunoregulators, limiting the extent of inflammatory reactions under all but the most intense situations. It is of interest that Katsumata et al [78] have shown that inflammatory cytokines, such as IL-1 or TNF-α, can upregulate the mRNA for the human endogenous retrovirus HERV-R (ERV3) in human vascular endothelial cells since we had previously reported that endothelial cells treated for 4 h with IL-1 released anti-inflammatory proteins which reacted with anti-p15E antibodies [85]. We have recently found that rabbit antiserum raised against the MN10021-homologous region of ERV3 recognizes retroviral p15E and that rabbit antiserum raised against p15E recognizes ERV3 (unpublished observations) suggesting that the highly conserved regions of both p15E and ERV3 might share not only sequence homology (∼60%) but conformational homology as well.

Although our data initially suggested that the anti-inflammatory/vasoprotective effects of MN10021 might be due to its induction of NO, additional studies have now shown that NO induction by these peptides is not required for their *in vitro* vasoprotective effects. Further studies on these peptides will be necessary to determine the mechanism(s) involved in their vasoprotection and whether vasoprotection is the basis for their potent anti-inflammatory effects.

## Conclusions

A transmembrane domain from retroviruses, including those from humans, has been conserved over a considerable time period on the evolutionary scale. This suggests that these sequences possess highly significant *in vivo* activities either of the type shown here or perhaps others as yet unknown. Peptides corresponding to these conserved regions have a variety of immunosuppressive, anti-inflammatory and vasoprotective effects. The exact role these sequences play in normal homeostasis and disease pathogenesis will be the object of further studies.

## Supporting Information

Methods S1 **The methods used to generate the data concerning the effects of MN10021 and its control peptide, MN20050, on human mononuclear cells as presented in** **Table 1** **are described in detail in Methods S1.**
(DOCX)
