# Supplementary material for: Anti-Inflammatory and Vasoprotective Activity of a Retroviral-Derived Peptide, Homologous to Human Endogenous Retroviruses: Endothelial Cell Effects
Source: PLoS One. 2012 Dec 20;7(12):e52693. doi: 10.1371/journal.pone.0052693 (PMC3527569; doi:10.1371/journal.pone.0052693)
Supplement: Methods S1 — The methods used to generate the data concerning the effects of MN10021 and its control peptide, MN20050, on human mononuclear cells as presented in Table 1 are described in detail in Methods S1. (DOCX) [file pone.0052693.s001.docx]

**Supplemental Methods**

Cytokine Assays: One-hundred µL of PBMC (2 x 10^6^/ml in Complete Medium) were added to each well of a flat-bottom, 96-well tissue culture plate. To each well was then added 50 µL of media or peptide (4X desired final concentration; in Complete Medium) and 50 µL of lipopolysaccharide (LPS; *S. typhosa*; Sigma, St. Louis, MO; 400 ng/ml Complete Medium). Each peptide was tested in triplicate at 5 log_10_ concentrations ranging from 100 µM to 10 nM. Plates were incubate 18-24 h at 37°C in 5% humidified CO_2_. Plates were then centrifuged and 100 µL of supernatant removed from each well and stored frozen at -80°C until assayed. Cytokines (TNF-α, IL-1β, IL-6, GM-CSF) were measured by sandwich ELISA assays (R & D Systems; Minneapolis, MN) using the manufacturer’s instructions. The released cytokine concentration was determined from a standard curve using recombinant cytokine provided with each kit. For each peptide the released cytokine concentration was plotted versus the peptide concentration and an IC_50_ (concentration resulting in 50% inhibition) was estimated from the resultant graph by determining the intersect of the line with the cytokine concentration representing 50% of the maximum observed with LPS alone.

Cytokine measurements of serum samples from MN10021-treated mice were performed by sandwich ELISA assays (R & D systems) according to the manufacturer’s instructions for use of its kits with sera samples.

Tissue Factor (TF): Monocyte-associated TF was quantitated in the following manner. PBMC were cultured in 48-well tissue culture plates as described below for PAF and LTB_4_ assays. At the end of the culture period (18-24 h) the supernatants were removed and stored frozen for cytokine assays. The wells of the plates were washed once with PBS and to each well was added 100 µL of Triton X-100 (10% [v/v] in H2O; Sigma, St. Louis, MO). The plates were placed on an orbital shaker for 12-18 h at 4°C and a speed of ~ 125 rpm. To each well was added 900 µL of sample buffer (10 mM PBS, pH 7.4, with 1% [w/v] BSA). The contents of each well were mixed well and removed for quantitation of TF using an IMMUBIND® Tissue Factor ELISA kit (American Diagnostica; Stamford, CT) according to the manufacturer’s instructions. Samples were diluted 1:10 in sample buffer prior to being assayed. For each peptide the TF concentration was plotted versus the peptide concentration and an IC_50_ (concentration resulting in 50% inhibition) was estimated from the resultant graph by determining the intersect of the line with the TF concentration representing 50% of the maximum observed with LPS alone.

Platelet Activating Factor (PAF) and Leukotriene B_4_ (LTB_4_) Assays: PBMC were prepared as described above and adjusted to 3.3 x 10^6^ cells/ml in RPMI-1640 medium containing 5 mg/ml HSA (Sigma, St. Louis, MO). To each well of a 48-well tissue culture plate was added 0.3 ml of cell suspension. The plate was incubated for 2 h at 37°C and 5% CO_2_ to allow cells to adhere. The wells were then washed 3X with HHBSS (Hank’s Balanced Salt Solution with 5 mg/ml HSA) at room temperature (RT). One-hundred seventy-five µL of HHBSS or test material in HHBSS was then added to each well and the plates incubated for 1 h at 37°C and 5% CO_2_. Samples were set up in quadruplicate. To each of duplicate wells for each sample was then added 175 µL of HHBSS or HHBSS containing 600 µg/ml of Zymosan (Sigma, St. Louis, MO). The plates were then incubated for 90 min at 37°C and 5% CO_2_. The plates were centrifuged and supernatants removed for quantitation of PAF or LTB_4_ according to the kit manufacturer’s (Amersham Pharmacia; Piscataway, NJ) instructions. The released PAF or LTB_4_ concentration was determined from a standard curve using standards provided with each kit. For each peptide the released PAF or LTB_4_ concentrations were plotted versus the peptide concentration and an IC50 (concentration resulting in 50% inhibition) was estimated from the resultant graph by determining the intersect of the line with the PAF or LTB_4_ concentration representing 50% of the maximum released by Zymosan alone.

Monocyte and Neutrophil Chemotaxis: For monocyte chemotaxis, PBMC were prepared as described above but washed and resuspended to 2 x 10^6^/ml in HBSS (10 mM HEPES, 0.5% BSA [low-endotoxin; Sigma, St. Louis, MO], pH 7.0). The percentage of monocytes in the cell preparation was determined by myeloperoxidase staining of fixed, cytospin-prepared slides and usually ranged between 15-25%. For neutrophil chemotaxis, neutrophils were prepared as described above and routinely contained 97-99% neutrophils. Effects of test materials on chemotaxis were measured using 48-well micro-chemotaxis chambers (Neuro-Probe; Cabin John, MD). Briefly, to 450 µL of cell suspension in a polypropylene centrifuge tube was added 50 µL of buffer or test material. Each sample was tested in triplicate. The tubes were then incubated for 60 min at 37°C in a shaking water bath. The cell suspensions were then added to the top wells of the chemotaxis chambers and a chemoattractant (n-formyl-methionyl-leucyl-phenylalanine; FMLP; Sigma, St. Louis, MO) in buffer was added to the bottom wells of two separate chambers at a final concentration of 10^-8^ or 10^-9^ M respectively. The upper and lower compartments of the chemotaxis chambers were separated by polycarbonate filters (5 micron for monocytes, 3 micron for neutrophils; Millipore; Bedford, MA). The chambers were incubated 90 min at 37°C in a humidified chamber, the wells of the upper chamber aspirated, and the chambers disassembled quickly and the filters stained using Diff-Quik (American Scientific Products; McGaw Park; IL). The stained filters were mounted on slides and the number of cells migrating though the filters determined under 600X magnification for 5 fields/well. Inhibition of chemotaxis was determined using that slide for which the positive control (untreated cells; either 10^-8^ or 10^-9^ M FMLP) gave the strongest response and was calculated in the following manner:

$$\% Inh=\frac{(\# cells migrating to FMLP-bkgd) untx-(\# cells migrating to FMLP-bkgd) tx}{(\# cells migrating to FMLP-bkgd) untx}$$

where: *Inh* = inhibition; *bkgd* = background; *untx* = untreated; and *tx* = treated.

Peptides were tested at 5 log_10_ dilutions ranging from 100 µM to 10 nM, inhibition plotted versus concentration and an IC_50_ (concentration resulting in 50% inhibition) determined from the resultant graph.

Monocyte Adhesion to Endothelial Cells: To each well of a 96-well tissue culture plate was added 100 µL of HUVEC at 2 x 10^4^ cells/ml. The plates were then incubated 48 h at 37°C and 5% CO_2_. PBMC, prepared as above, were washed once in serum-free RPMI-1640 medium. Calcein-AM (Sigma; St. Louis, MO) was added to a final concentration of 10 µg/ml, the cells incubated 30 min at 37°C, and then washed once with serum-free RPMI-1640. The cells were then washed an additional time with Complete Medium and resuspended to a concentration of 3 x 10^6^/ml in Complete Medium. For each concentration of compound to be tested, 200 µL of labeled PBMC was incubated with 200 µL of test compound (2X desired final concentration) in polypropylene tubes. Similarly, for each concentration of compound to be tested, 50 µL of medium was removed from a well of the plated HUVEC and 50 µL of medium containing the test compound (2X desired final concentration) was added. Both tubes and plates were incubated 1 h at 37°C in 5% CO_2_. All peptides were tested at log_10_ concentrations of 10 nM to 100 µM. To each well or tube was then added 10 or 100 µL respectively of either media or TNF-α (5 ng/ml; R & D Systems; Minneapolis, MN) and the tubes and plates incubated an additional 6 h at 37°C in 5% CO2. For each test condition, 100 µL of treated PBMC was added to a corresponding well in the plate and the plate incubated an additional 30 min at 37°C in 5% CO_2_. The plate was then read on a fluorescent spectrophotometer with filter settings of λ = 485exc/530emi. The plate was then inverted, the media removed by blotting, the plate sealed with Parafilm M (American National Can; Menasha, WI) and then centrifuged in the inverted position for 5 min at 250 x g. The plate was blotted to remove any media and read again on the fluorescent spectrophotometer. Percent of Maximum Adhesion (% *Max. Adh.)* was calculated as:

$$\% Max. Adh. = \frac{Absorbance in Treated Wells}{Absorbance in Untreated Wells} \times100$$
